# A 77-Year-Old Male with a Rapid Change in Mental Status

**DOI:** 10.5811/cpcem.7213

**Published:** 2024-06-14

**Authors:** Andrew Piner, Spencer S. Lovegrove, Laura J. Bontempo, T. Andrew Windsor

**Affiliations:** * University of Maryland Medical Center, Baltimore, Maryland; † University of Maryland School of Medicine, Department of Emergency Medicine, Baltimore, Maryland

**Keywords:** *air embolism*, *stroke*, *CPC*

## Abstract

A 77-year-old male who presented to the emergency department with generalized weakness and worsening chronic dysphagia was diagnosed with pneumonia. Shortly after receiving vascular access for his treatment, he had a rapid change in his mental status and neurological examination.

Population Health Research Capsule What do we already know about this clinical entity?

*Air embolism is a rare but potentially life-threatening complication of various procedures, including central line placement.*

What makes this presentation of disease reportable?

*This patient experienced a cerebral air embolism but did not have a patent foramen ovale, and the air embolism remained within the venous system.*

What is the major learning point?

*Although rare, clinicians should consider air embolism for a patient who has an unexpected decline in their condition shortly after an intravascular procedure.*

How might this improve emergency medicine practice?

*This case discusses treatment of this serious condition, including bedside treatment and hyperbaric oxygen therapy.*

## CASE PRESENTATION (Dr. Piner)

A 77-year-old male presented to the emergency department (ED) for generalized weakness as well as worsening chronic dysphagia. He was accompanied by his wife, who assisted in providing the history. His past medical history included diabetes, hypothyroidism, a thyroid goiter, right internal jugular (IJ) thrombus for which he was taking anticoagulation, and cirrhosis. His surgical history was significant for remote bilateral percutaneous nephrostomy tube placement, years prior. Three weeks preceding this hospital visit, he had been admitted and discharged from the same hospital after receiving a thoracentesis with drainage of a simple transudative pleural effusion. This incompletely relieved his hypoxia, and he was discharged on three liters per minute (L/min) of oxygen via nasal cannula.

Since returning home, the patient thought his breathing had slightly improved, but he had difficulty completing his daily activities. He had a poor appetite and when he did eat, he experienced dysphagia from his goiter, which he felt was getting worse. He reported general malaise and chills. He did not feel short of breath on exertion, as he had during his last hospitalization, and he was not experiencing any chest discomfort. He denied any nausea, vomiting, constipation, diarrhea, dysuria, or urinary changes. He was able to ambulate but had difficulty doing so due to his weakness.

The patient was recovering from alcohol use disorder and denied having any alcoholic drinks in multiple years. He had a remote, five pack-year tobacco history but did not smoke any longer. He denied any use of illicit drugs. His wife oversaw his medications, which had not changed since his hospital discharge. He took metoprolol, spironolactone, levothyroxine, and apixaban. He had no known drug allergies.

The patient’s vital signs revealed an oral temperature of 100.4° Fahrenheit (38° Celsius), heart rate of 90 beats per minute, a blood pressure of 90/40 millimeters of mercury (mm Hg), a respiratory rate of 15 breaths per minute, and his oxygen saturation was 94% on 3 L/min via nasal cannula. He weighed 64 kilograms (142 pounds) and was 170 centimeters tall (5 feet 7 inches) with a body mass index of 22.1.

On physical examination, the patient was alert and oriented to person, place, time, and events. He was able to speak in full sentences without dyspnea. He did not appear in acute distress. He had evidence of a large goiter and denied tenderness or the sensation of it expanding or changing. His breath sounds were diminished at the right lower base with scattered rhonchi and an occasional cough. His heart sounds were regular without murmurs, rubs, or gallops. His abdomen was large, protuberant, and had a soft fluid wave that could be appreciated. It was not increased in size per his wife. There was no guarding or tenderness. He had trace pedal edema without lateralizing swelling. He moved all extremities without localizing weakness. Although he reported weakness, his strength was 5/5 symmetrically in his upper and lower extremities. His cranial nerves (CN) II–XII were intact. His Glasgow Coma Scale (GCS) score was 15/15 – Eye 4, Verbal 5, Motor 6. His pupils were equal, round, and reactive to light. He was not ambulated in the room but had been seen earlier moving from a hospital wheelchair to the stretcher with assistance. There was no skin rash or joint swelling.

**Image 1.**
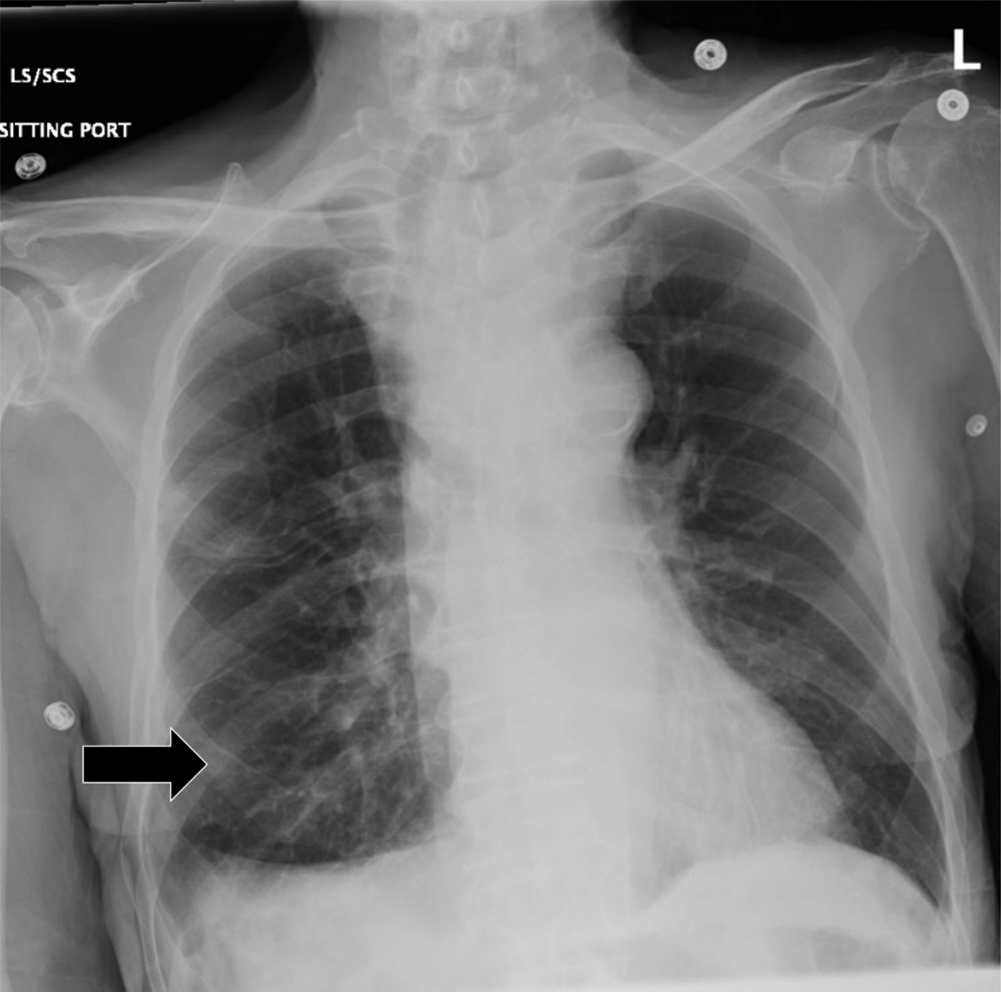
Initial chest radiograph showing an opacity in the right midlung (black arrow) in a 77-year-old man before he experienced a rapid change in mental status.

Initial laboratory results are shown in Table. An electrocardiogram (ECG) was performed and showed a sinus rhythm without ectopy or ST-segment changes and was unchanged from his previous hospitalization. Chest radiography (CXR) was obtained, shown in Image 1. The radiologist’s interpretation was “[p]atchy opacity in the right lower lobe suggestive of pneumonia in the correct clinical setting. Goiter redemonstrated when compared to previous. Normal cardiac findings.” A point-of-care ultrasound (POCUS) of the heart showed no pericardial effusion, no suggestion of right ventricle strain, no volume overload, and a normal left ventricle.

**Table.** Initial laboratory results of a 77-year-old man with a rapid change in mental status.

Test name	Patient value	Reference range
Complete blood count		
Hemoglobin	10.4	11.9–15.7 g/dL
Hematocrit	32.3	35.0–45.0%
White blood count	18.2	4.5–11 K/mcL
Platelet	187	153–367 K/mcL
Neutrophils	72.5	42.6–74.5%
Lymphocytes	10.3	20.8–50.5%
Monocytes	16.2	2.0–10.3%
Eosinophils	0.2	0.9–2.9%
Complete metabolic panel		
Sodium	132	136–145 mmol/L
Potassium	4.0	3.5–5.1 mmol/L
Chloride	102	98–107 mmol/L
Bicarbonate	24	21–30 mmol/L
Glucose	147	70–99 mg/dL
Blood urea nitrogen	21	7–17 mg/dL
Creatinine	0.74	0.52–1.04 mg/dL
Calcium	7.8	8.6–10.2 mg/dL
Magnesium	1.5	1.6–2.6 mg/dL
Phosphorus	2.9	2.5–4.5 mg/dL
Total protein	5.2	6.3–8.2 g/dL
Total bilirubin	1	0.3–1.2 mg/dL
Aspartate aminotransferase	46	14–36 units/L
Alanine aminotransferase	21	0–34 units/L
Alkaline phosphatase	82	38–126 units/L
Coagulation		
Protime	15.9	12.1–15.0 seconds
International normalized ratio	1.3	0.8–1.1
Partial thromboplastin time	45	25–38 seconds
Fibrinogen	280	200–400 mg/dL
Thyroid		
Thyroid stimulating hormone	0.01	0.4–4 mIU/L
Free Thyroxine	6.6	0.8–1.8 ng/dL
Additional tests		
Lactate	1.7	0.5–2.2 mmol/L
Ammonia	9	9–30 mcmol/L
Troponin I	0.02	<0.06 ng/mL

*dL*, deciliter; *g*, grams; *K*, thousands; *mcL*, microliter; *mcmol*, micromole; *mg*, milligram; *mIU*, milli-international units; *mmol*, millimole; *ng*, nanogram; *L*, liter.

Before treatment for pneumonia could be started, the nurse alerted the physician to the loss of vascular access. There were no reliable peripheral veins seen via ultrasound; so, central access was obtained in the left IJ vein via ultrasound guidance while the patient was in a semi-reclined position. The wire was confirmed in the vein and removed. The physician instilled a bolus of agitated normal saline, confirming the central access was in the venous system by visualizing turbulent flow in the right atrium with POCUS. The line was sutured and dressed.

Fifteen minutes later, the physician was called to the room as the patient’s oxygen requirement increased and he was less responsive. Repeat physical examination revealed new tachycardia with a mean arterial pressure of 59 mm Hg. The patient’s respiratory rate was 20, and his oxygen saturation remained 94% on 15 L/min via a non-rebreather mask. Repeat POCUS showed unchanged cardiac function and no evidence of pneumothorax. The patient was unable to follow commands, his eyes opened only to pain, and he muttered incomprehensible words. He was noted to withdraw to pain on the right arm and leg, the left arm was flaccid, and the left leg was flexed. His new GCS was 8/15 – Eye 2, Verbal 2, Motor 4. His left eye was midline, but his right eye was noted to deviate laterally and inferiorly.

**Image 2.**
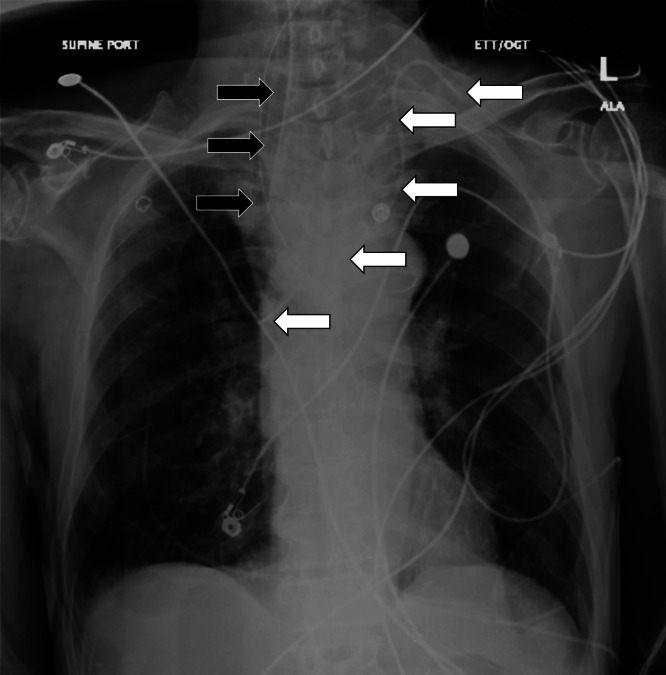
Repeat chest radiograph of a 77-year-old man after a rapid change in mental status showing the placement of an endotracheal tube (black arrows) and left internal jugular central line (white arrows).

The patient was intubated, and a confirmatory CXR demonstrated interval changes of a new left IJ central line catheter and an endotracheal tube that both appeared adequately positioned as seen in Image 2.

A test was performed, and a diagnosis was made.

## CASE DISCUSSION (Dr. Lovegrove)

This is a case of an elderly gentleman who acutely decompensated in the ED after initially presenting due to worsening weakness and dysphagia. When there is a patient who acutely decompensates in the ED, as this patient did, it is important to consider whether this was the result of the patient’s natural disease course, something that happened in the ED (iatrogenic), or even a little bit of both.

The patient’s initial complaint is worsening dysphagia as well as weakness. He has an extensive medical history that includes diabetes, hypothyroidism, goiter, right IJ thrombus, and anticoagulant use. His physical exam is notable for multiple abnormalities including fever, hypotension, abnormal breath sounds in his right lung, and ascites. It is also important to point out that his neurologic exam is normal, including full strength in all extremities.

Looking over the labs and imaging, I do not believe they provide a significant amount of new information but could be used to remove some differentials. The patient’s chemistry had some slight abnormalities but nothing unexpected given his comorbidities. His coagulation studies were consistent with someone with cirrhosis. There were some abnormalities in his thyroid function panel, but I do not think they explain his acute change. We also do not know the timing of his levothyroxine dose, and his thyroid stimulating hormone indicates that he has likely been taking it. The etiology of hematuria is unclear, but his urine is otherwise without any sign of infection. His complete blood count does show leukocytosis as well as mild anemia. Lastly, his CXR is subtle, but an opacity in the right midlung, as interpreted by radiology, would be consistent with his abnormal breath sounds.

Based off the history, physical exam, and diagnostic studies, the patient’s initial presentation was concerning for sepsis, potentially from pneumonia associated with aspiration or due to spontaneous bacterial peritonitis (SBP). However, I do not believe these issues alone would necessarily progress to cause such an abrupt change in mental status and neurologic function. Was his dysphagia being caused simply by mass effect of his goiter, or was it something else? Could there be another underlying etiology that was exacerbated by the patient’s septic state?

Once he decompensated, his exam changed, and the differential expanded. He now had a GCS of 8 with gaze deviation of his right eye “down and out” to the right, but his left pupil is midline. He also had a flaccid left arm and his left leg remained flexed. His right arm and leg withdrew to pain. I considered the following differentials, grouped by category, based off his initial presentation and chief complaint as well as the events of his ED visit, as it is unclear whether they are related.

*Autoimmune:* For a potential autoimmune cause, I did consider potentially undiagnosed myasthenia gravis (MG). He had a known history of goiter and thyroid disease, which does have an association with developing MG. Patients with MG can present with asymmetric muscle weakness that commonly involves the extraocular muscles. Asymmetric presentations can mimic a stroke. Perhaps his worsening dysphagia and weakness were being caused by MG, and he rapidly deteriorated in the ED due to his sepsis. He may have had unrecognized respiratory muscle compromise and may have become hypercapnic, causing his altered mental status. This is unlikely since he had no evidence of any weakness on his initial exam, but it could not readily be ruled out by the results I was given, so I kept it on the differential.

*Infectious:* As we discussed earlier, it is possible that he has pneumonia or SBP; however, I do not think they would cause these focal neurologic abnormalities. His blood pressure is on the lower end, potentially due to sepsis, but I do not believe he would have suffered a watershed infarct causing these specific abnormalities. Similarly, I considered meningitis or encephalitis due to his fever and altered mental status, but again I do not think they explain his rapid deterioration and focal deficits. He also did not complain of any neck pain or headache prior to his decompensation.

*Cardiac:* Arrythmia is always a potential cause of acute decompensation. I would have loved to have seen a repeat ECG or rhythm strip at the time of decompensation, but there was no mention of him being particularly tachycardic or bradycardic with his repeat vitals, so I feel this is unlikely. I considered the possibility of acute heart failure or cardiac tamponade causing hypotension and subsequent poor cerebral perfusion and altered mental status, but the point-of-care echocardiogram remained essentially normal.

*Pulmonary:* I considered pneumothorax as a complication of central line placement as a cause of the acute respiratory decompensation; however, this was not seen on CXR after placement, and it should not cause focal neurologic deficits.

*Endocrine/metabolic:* Hypoglycemia and hyponatremia can both cause acute altered mental status; however, the patient’s labs showed no evidence of hyponatremia or hypoglycemia, and the treatments he received in the ED should not have caused them. Uremia and hyperammonemia were ruled out in his chemistry panel. I considered thyroid storm due to his fever and elevated free T4; however, this patient’s T4 is presumably coming from his levothyroxine. Without acute changes to his regimen or suspected overdose of his medications, I felt this was unlikely.

*Toxicological:* There is no history of abnormal exposures. He was started on antibiotics, as well as norepinephrine. Some antibiotics, such as cefepime, can produce neurologic abnormalities such as seizures, but it would be unlikely to occur this rapidly after initial administration. He also presumably received lidocaine as part of his central line placement. Lidocaine toxicity can cause altered mental status and seizure. Often, when administered as part of central line placement, about 5–10 milliliters (mL) of 1% lidocaine is used, and precautions are taken to avoid vascular injection. This dosage should not be high enough to cause acute lidocaine toxicity.

*Hematologic:* I did consider thrombotic thrombocytopenic purpura (TTP) as a potential cause. Infection can prompt the development of TTP and could potentially explain the patient’s neurologic deficits hematuria and proteinuria. However, I eliminated this from the differential as he had only mild anemia and no significant thrombocytopenia or evidence of renal failure.

*Neurologic:* The patient’s acute abnormalities that developed in the ED were primarily neurologic, which means this is the organ system I considered the most heavily. The new physical exam is interesting because classically in a hemispheric stroke there is conjugate gaze deviation, with both eyes looking toward the lesion. In seizure, the conjugate gaze deviation is classically away from the lesion. The disconjugate gaze, as well as the left arm weakness involving the opposite side as the gaze deviation, led me away from a large hemispheric ischemic or hemorrhagic stroke. While considering diagnoses that could cause unilateral gaze deviation, I again considered muscular weakness being caused by a disorder at the neuromuscular junction, such as MG; however, I also considered ischemic or compressive lesions to CN III. Cavernous sinus thrombosis has been known to cause isolated cranial nerve palsies, including CN III, IV, V, and VI. This patient did have a history of thrombosis with a previous IJ thrombus, but he was currently taking an anticoagulant and had not been complaining of headache.

The patient’s symptoms were fairly consistent with a stroke in the midbrain and Weber syndrome, which is described as having an ipsilateral CN III palsy with contralateral hemiplegia. However, those patients generally have a relatively normal mental status, whereas our patient had a GCS of 8. It could be possible that he had multiple areas of infarct or disease that are making it difficult to pinpoint the exact location of his lesion or lesions.

After considering these differentials and the results that were available, I ultimately narrowed the differential down to three final diagnoses. An undiagnosed neuromuscular junction disorder, such as MG, would potentially explain his multiple deficits and incorporate his original chief complaint. My last two differentials are similar in that they are both iatrogenic and related to his central line placement involving different types of emboli. He decompensated shortly after the placement of the line; so, a potential iatrogenic cause needed heavy consideration.

He had a known history of prior right IJ thrombus; so, perhaps a central line was placed through a new, undiagnosed left-sided IJ thrombus and caused a shower of embolic thrombi. However, for this to have caused a stroke instead of a pulmonary embolism, he would have also needed to have a patent foramen ovale to allow shunting to the arterial circulation. If the clinician used ultrasound during the placement of the central line and the patient has been taking his anticoagulation, hopefully placement through a thrombus would have been avoided. This right-to-left cardiac shunting could also have been detected during the bolus of agitated saline visualized for line confirmation on point-of-care echocardiogram, and there was no mention of this.

For my final differential, I considered air embolism. A shower of air emboli to the midbrain, as well as multiple other areas of the brain, could explain his focal neurologic deficits as well as his suppressed mental status. This is a rare but possible complication of central line placement, and retrograde venous flow of air is possible due to buoyancy if the patient is upright. It was noted that the patient was semi-reclined during placement, rather than in the preferable Trendelenburg position, which can be protective against air embolus. Like a thrombus, air emboli could also be transmitted to the arterial circulation if this patient had an undiagnosed patent foramen ovale.

Ultimately, I did not choose undiagnosed MG since his initial strength testing was completely normal. With the patient already taking anticoagulation, a new thrombus is less likely; so, I opted to choose air embolism as my final diagnosis as it makes the most sense with the timing after central line placement and the multiple different neurologic deficits. My test of choice from the ED would be computed tomography (CT) of the head.

## CASE OUTCOME (Dr. Piner)

**Image 3.**
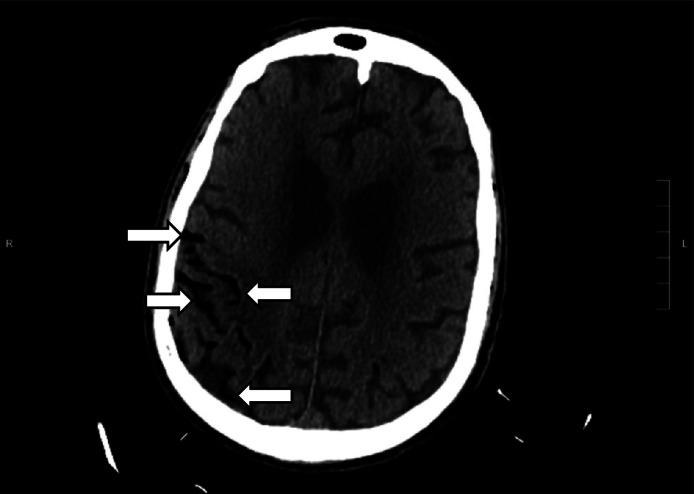
Computed tomography of the brain of a 77-year-old man after a rapid change in mental status showing extensive venous gas (white arrows) in the right parieto-occipital region.

The patient underwent an emergent CT of the head (Image 3) due to the change in mental status. The radiology impression of the CT revealed “extensive venous gas, which may indicate gas embolus with possible evolving infarction in the right parietal region. No hemorrhage or shift. Further evaluation with magnetic resonance imaging [MRI] may be useful. Large goiter.” Immediately the team assessed the patient’s central venous catheter and found an uncapped line. After the air was withdrawn from the line, the line was capped. The patient was transferred to the intensive care unit at our hospital for further management and a hyperbaric medicine consultation. He underwent a hyperbaric oxygen therapy treatment with resolution of the gas on the repeat CT head. A follow-up MRI revealed multifocal infarcts in multiple vascular territories.

In the following days, the patient’s mental status unfortunately never improved. Palliative care discussions with the patient’s family indicated that he would not want to have any further artificial prolongation of life without any meaningful neurologic functioning, and the team transitioned his care to comfort measures only. He was transferred to hospice care and died surrounded by his family.

## RESIDENT DISCUSSION (Dr. Piner)

Air embolism represents a rare phenomenon when air enters either arterial or venous circulation with subsequent obstruction of the vasculature, preventing distal blood flow. The condition requires that there be a direct connection between the vasculature and the gas source. This can be due to vascular trauma prompting gas entry or direct entry from placement of an intravascular catheter. Many of the cases of air embolism involve a preventable iatrogenic process, prompting the Centers for Medicare and Medicaid Services at one point to classify it as a “never event” along with other preventable conditions such as falls, retained surgical objects, and incompatible blood transfusion.^1^

The rate of air embolism following a central line manipulation (insertion, drug delivery, or removal) is estimated to be between 0.3–2%.^2^ Considering how commonplace central access insertion is, this could involve a significant level of morbidity and mortality. However, not all cases of air embolism are as dramatic or symptomatic as in this case. Most air emboli will be asymptomatic and unrecognized, as their volume and rate of accumulation may be miniscule. The lethal dose depends on location of the obstruction, volume, and rate of administration, but lethal doses range between 3–5 milliliters (mL) per kilogram or about 200–300 mL in adults.^3^

Other etiologies of air embolisms involve blast injuries, barotrauma, and direct vascular trauma. Surgery has been associated with air embolism, particularly neurosurgery, where the incidence of air embolism has been reported to be higher when the patient was undergoing an open craniotomy in a seated or semi-seated position.^4^

The key for air embolism is prevention. This is particularly important to remember when obtaining vascular access, manipulating existing catheters, or other high-risk surgical procedures. High-risk vascular access involves large-bore catheters, emergent placement, access site above the level of the heart, and pressure-infused fluids (eg, arterial line or rapid infusion machine). Care can be taken to properly position patients when placing catheters and placing the vascular access point at or above the level of the heart. Lines should be immediately capped or clamped to prevent direct air entry. Although debated, asking your patient to exhale during removal may prevent negative intrathoracic pressure, which enhances venous return and can pull air in if there is an entry portal. Additionally, pressure should be held after the catheter is removed in all cases.^5^

Prompt recognition is crucial for treatment. Diagnosis should be made rapidly by first having a high index of suspicion and then followed by treatment. The location in the arterial or venous system, amount of gas, and the organ affected will dictate the management, as well as the signs and symptoms guiding the workup.

If the air embolism occurs in the pulmonary system, it could be expected to behave like a thrombotic pulmonary embolism. However, if there is a connection between the venous/arterial side, as in the context of a patent foramen ovale, then venous gas could traverse and cause symptoms of arterial ischemia. Our patient had cerebral venous gas, likely as a result of retrograde flow with the patient in an upright position.

A POCUS can be performed to look for a gas bubble in the right ventricle as well as alternative explanations for the change in a patient’s hemodynamics (for example, pneumothorax or pericardial tamponade). Transesophageal echocardiogram (TEE) is more sensitive for detection; however, there is limited access to this method, and it may be more applicable in the operating room. Some high-risk procedures use a TEE intraoperatively to proactively monitor for such events. Diagnostic measures should be ordered based on the site of the suspected embolism. These may commonly include a troponin, ECG, lactate, or renal function. Imaging will help confirm the diagnosis; CT angiography is the most likely diagnostic modality, although air can sometimes be seen on plain films.

If an air embolism is suspected, the site of air entry should be covered if open, pressure held, and any offending actions (line insertion, insufflation, pressure infusion) should be stopped. The next step involves positioning the patient to trap gas in the venous system and prevent it from causing complete cardiovascular collapse in the form of an air trap. The patient should be placed in the left lateral decubitus position in the Trendelenburg position (head down). This is called the Durant maneuver. This allows for blood to still pass into the pulmonary artery while displacing and hopefully trapping the air bubble away from the right ventricular outflow tract. It also prevents right-to-left traversing of the gas bubble.^5^

Treatment involves supportive care of the organ affected. Supplemental oxygen should be applied via a non-rebreather mask. If an air embolism is suspected and there is a central catheter in the superior vena cava, then one can attempt to aspirate blood from the distal tip in hopes of suctioning the air embolism.

If these measures fail to alleviate the cardiovascular collapse, then venous-arterial extracorporeal membrane oxygenation could provide rescue therapy as a bridge to definitive care. Interestingly though, this procedure itself has a high risk of causing an air embolism if there is any air in the catheters when they are hooked to the circuit. The gold standard involves hyperbaric oxygenation therapy with earlier treatment preferred; however, cases have been successfully treated after 24 hours of symptoms, as the availability of dive resources may vary widely among institutions and involve prolonged and careful transport.^6^

## FINAL DIAGNOSIS

Cerebral air embolism secondary to central line complication.

## KEY TEACHING POINTS

• Rapid changes in a patient’s clinical stability may be related to progression of the known presenting disease or a new process entirely.
• Central venous catheter placement is not a benign procedure, and given its frequent use, care should be taken to avoid complications.
• Cerebral air embolism is caused by air entry directly into the vascular space, which may present as a change in a patient’s neurological examination.
• Prevention is key for air embolisms; however, if one occurs then oxygen, Durant maneuver, and hyperbaric therapy are the cornerstones of therapy.
